# Analytical multi-soliton solutions of a (2+1)-dimensional breaking soliton equation

**DOI:** 10.1186/s40064-016-2403-2

**Published:** 2016-06-27

**Authors:** Shao-fu Wang

**Affiliations:** College of Electrical and Electronic Engineering, Department of Electronics, Anhui Science and Technology University, Bengbu, Fengyang, 233100 Anhui People’s Republic of China

**Keywords:** Breaking equation, Darboux transformation, Weierstrassp function, Multi-soliton

## Abstract

The analytical solutions for a (2+1)-dimensional breaking solution equation is proposed in this paper by using mapping and projective method darboux transformation, and Some exact propagating solutions are constructed for this Breaking equation, and the *M* × *N* multi-soliton could be obtained by using Weierstrassp function and setting the perfect parameters. The potential application of breaking Soliton equation will be of great interest in future research.

## Background

In recent years, many research have studied soliton and its evolvements in nonlinear equations via kinds of method (Zarmi 2014; Chen and Ma 2013; Ashorman 2014; Zhang and Chen 2016; Meng and Gao 2014; Mohamed 2016; Liu and Liu 2016; Jiang and Ma 2012; Guo and Hao 2013; Dou et al. 2007). Vertex dynamics in multi-soliton solutions and some new exact solution of breaking equation are studied in Zarmi (2014) and Chen and Ma (2013), the methods of Multi-soliton Solutions are given in Ashorman (2014), Zhang and Chen (2016), Meng and Gao (2014), Mohamed (2016), Liu and Liu (2016), Jiang and Ma (2012), Guo and Hao (2013), Dou et al. (2007), Zuo and Gao (2014), Huang (2013), Liu and Luo (2013), Côtea and Muñoza (2014), Xu and Chen (2014), Hua and Chen (2014) and Zhang and Cai (2014), complex solutions for the [BLP System are proposed in Ma and Xu (2014)], and these so-called new solutions is identical to the universal formula in Doungmo Goufo (2016), Atangana and Doungmo Goufo (2015), Gao (2015a, b, c, d), Xie and Tian (2015), Sun and Tian (2015) and Zhen et al. (2015).

The aim of this paper is to investigate the analytical solutions of the (2+1)-dimensional breaking equation by the mapping and Darboux transformation method. And the dynamical behaviours of (2+1)-dimensional breaking equation will be discussed in detail.

The structure of this paper is as follows: In second section, the (2+1) dimensional breaking equation is studied and its exact solutions are derived. And properties of this breaking equation will be investigated. In third section, influence of the parameters which are related to the analytical solution will also be discussed. Finally, the conclusion is drawn in fourth section.

## (2+1)-dimensional breaking equation and exact solution

Consider (2+1)-dimensional breaking equation as follows (Dou et al. 2007):

1 $$ u_{t} + bu_{xxy} + 4bv_{x} + 4bu_{x} v = 0 $$

In which, the functions of $u(x,y,t)$ and $v(x,y,t)$ are corresponding physical fields and set

2 $$ u_{y} = v_{x} $$

In which b is the arbitrary function, substituting Eq. (2) into Eq. (1), the analytical solution of Eq. (1) could be gotten as follows:

3 $$ \left\{ {\begin{array}{*{20}l} {u = \frac{3}{2}(\ln f)_{xx} + u_{0} } \hfill \\ {v = \frac{3}{2}(\ln f)_{xy} + v_{0} } \hfill \\ \end{array} } \right. $$

4 $$ f = a_{0} + a_{1} p(x) + a_{2} q(y,t) + a_{3} p(x)q(y,t) $$

In which, $u(x,y,t)$ is seed solution of equation, $u_{0} = a(x),v_{0} = 0$, the parameters $a_{0} ,a_{1} ,a_{2} ,a_{3}$ are constants.

Substituting Eqs. (3) and (4) into Eq. (1), the analytical solution of (2+1) dimensional breaking equation could be gotten as follows (Dou et al. 2007)

5 $$ u = - \frac{3}{2}\left[ {\frac{{(a_{1} + a_{3} q)^{2} p_{x}^{2} }}{{(a_{0} + a_{1} p + a_{2} q + a_{3} pq)^{2} }} - \frac{{(a_{1} + a_{3} q)p_{xx} }}{{(a_{0} + a_{1} p + a_{2} q + a_{3} pq)}} + \frac{{bf_{xxx} + cf_{x} }}{{6bf_{x} }}} \right] $$

6 $$ v = \frac{3}{2}\left[ {\frac{{(a_{3} a_{0} - a_{1} a_{2} )p_{x} q_{y} }}{{(a_{0} + a_{1} p + a_{2} q + a_{3} pq)^{2} }}} \right] $$

## *M* × *N* multi-soliton

Define the Weierstrassp function as following

7 $$ X_{n + 1} = X_{n} + a^{n} \cos (2\pi b^{k} x) $$

8 $$ Y_{n + 1} = Y_{n} + a^{n} \cos (2\pi b^{n} y) $$

**Fig. 1:**
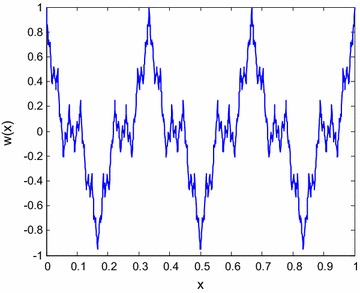
The response of Weierstrassp function

In which $0 < a < 1$, $ab \ge 1$, and when the parameters $a = 0.5,b = 3,n = 10$, its response is shown in Fig. 1 and we set

9 $$ \phi (x) = X_{n + 1} ; $$

10 $$ \varphi (y) = Y_{n + 1} ; $$

when $p = \phi (kx,k_{2} ,k_{3} )$, $q = \varphi (y + ct,k_{2} ,k_{3} )$, The Eq. (7) can be given as

11 $$ v = \frac{3}{2}\left[ {\frac{{(a_{3} a_{0} - a_{1} a_{2} )\phi_{x} \varphi_{y} }}{{(a_{0} + a_{1} \varphi + a_{2} \phi + a_{3} \phi \varphi )^{2} }}} \right] $$

in which

12 $$ \phi_{x} = \sqrt {4\phi^{3} - k_{2} \phi - k_{3} } $$

13 $$ \varphi_{y} = \sqrt {4\varphi^{3} - k_{2} \varphi - k_{3} } $$

**Fig. 2:**
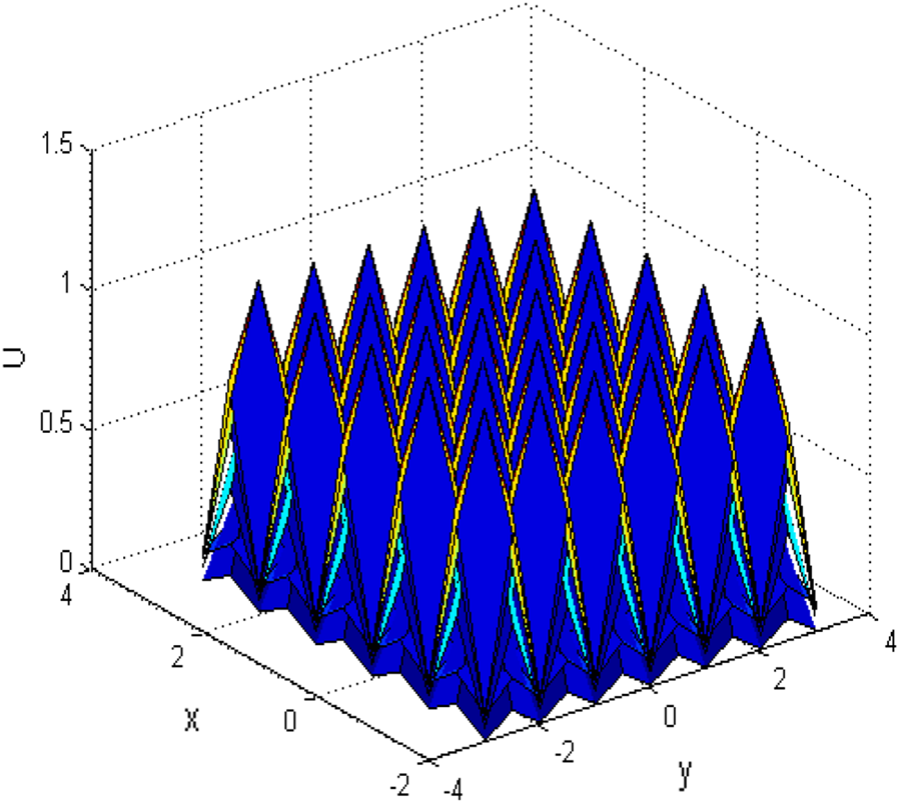
The 5 × 6 multi soliton structure

**Fig. 3:**
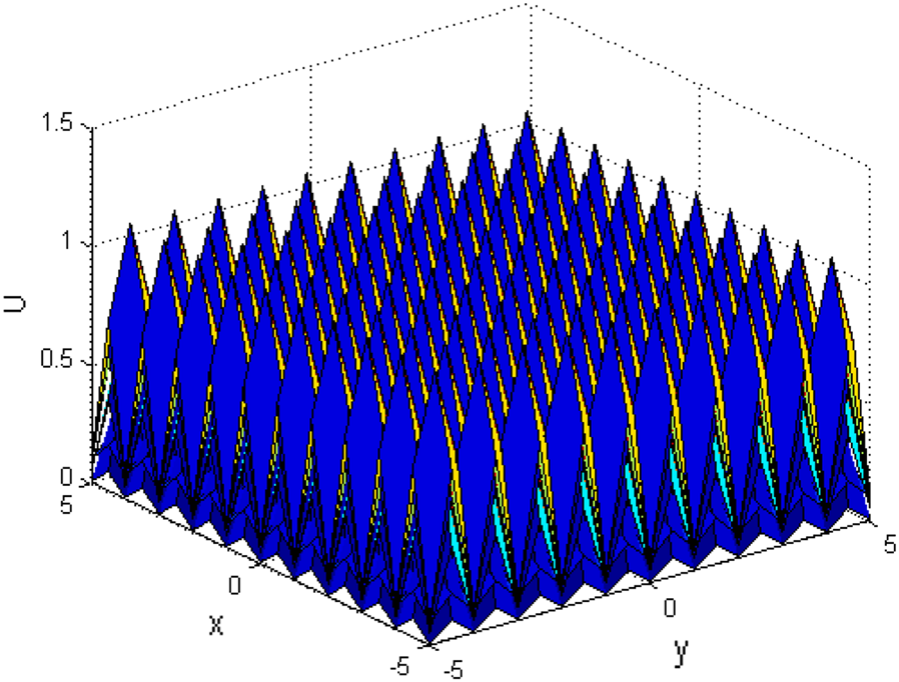
10 × 10 multi soliton structure

When the parameters $k,c,k_{2} ,k_{3}$, $n$ are selected as different constants, the $N \times M$ multi-soliton could be achieved according to Eq. (11), when the parameters $a_{0} = 4;a_{1} = 1;a_{2} = 1;a_{3} = 1;k = 0.6;k_{2} = 1$; $k_{3} = 1;c = 1;n = 5$, and $x \in [ - 3,3],y \in [ - 3,3]$, the $5 \times 6$ multi-soliton are shown in Fig. 2, and $x \in [ - 5,5],y \in [ - 5,5]$, the $10 \times 10$ multi-soliton structure are shown in Fig. 3.

From Figs. 2 and 3, it can be seen that multi-soliton could be obtained by selecting the Weierstrassp function.

## Conclusion

To summarize, we have constructed $M \times N$ multi-solutions of a (2+1)-dimensional breaking equation using Darboux transformation and mapping method. The analytical solutions which changes the shape of the solutions are explored and the derived analytical expressions of (2+1)-dimensional breaking equation can be used in communication system, and it is highly anticipated that this investigation on (2+1)-dimensional breaking equation may have wider application in various physical models.
